# Transferring health professionals social and cognitive skills: key findings from a qualitative investigation

**DOI:** 10.1186/s41077-025-00356-0

**Published:** 2025-05-21

**Authors:** Lotte Abildgren, Malte Lebahn-Hadidi, Christian Backer Mogensen, Palle Toft, Sune Vork Steffensen, Lise Hounsgaard

**Affiliations:** 1 Anesthesiology and Intensive Care Department, Odense University Hospital, Odense, Denmark; 2 Open Patient Data Explorative Network, OPEN, Odense University Hospital, Odense, Denmark; 3 Department of Clinical Research, University of Southern Denmark, Odense, Denmark; 4 Emergency Research Department, Hospital Sønderjylland, University Hospital of Southern Denmark, Odense, Denmark; 5 Centre for Research in Patient Communication, CFPK, Odense University Hospital, Odense, Denmark; 6 Centre for Human Interactivity, Department of Language and Communication, University of Southern Denmark, Odense, Denmark; 7 Institut for Regional Sundhedsforskning, Sygehus Sønderjylland Og Syddansk Universitet, Sønderborg, Denmark; 8 Simulation Center SimC, Odense University Hospital, Odense, Denmark; 9 Centre for Shared Decision Making, Lillebælt Hospital, Fredericia, Denmark; 10 Danish Institute for Advanced Study, University of Southern Denmark, Odense, Denmark; 11 Center for Ecolinguistics, South China Agricultural University, Guangzhou, China; 12 College of International Studies, Southwest University, Chongqing, China; 13 Institute of Nursing & Health Science, Ilisimartusarfik, University of Greenland, Nuuk, Greenland

**Keywords:** Cognitive skills, Healthcare, Health professional, Social and cognitive skills, Patient simulation, Safety Management, Simulation-based training, Social skills, Transfer, Psychology

## Abstract

**Background:**

Research shows that simulation-based training can increase knowledge and skills among pregraduate healthcare students, that simulation-based training of technical skills places the participants higher on the learning curve in practice, and that simulation-based training can improve participants’ social and cognitive skills. Nevertheless, how cognitive and social knowledge and skills are transferred into clinical practice competency remains unknown. This study aims to explore qualified in-hospital health professionals transfer of social and cognitive skills from a simulation-based training course to competency in everyday clinical practice.

**Method:**

A qualitativeResearch shows that simulation-based training can increase phenomenological-hermeneutic methodology and an ethnographic study investigate qualified health professionals’ social and cognitive skills transfer before, during, and after a simulation-based training course. The data collection comprises three phases: a clinical phase, a simulation-based training phase and a transfer phase; each phase is based on a subsequent analysis of the previous phase. Data consist of approximately 107 h of video recordings, field notes and reflections within the research team. Data are analysed with RICEA, a qualitative hybrid method of a Ricɶur-Inspired Analysis and Cognitive Event Analysis.

**Findings:**

The analysis reveals three key themes: *individual transfer of learning*, *intercollegiate transfer of learning* and *organisational transfer of learning*. The findings imply that transfer of social and cognitive skills happens on an individual and intercollegiate level. Still, transfer needs to be scaffolded on an organisational level so that cognitive and social knowledge becomes competency in clinical practice. Further, the findings imply that transferring social and cognitive skills needs a different focus from transferring technical skills. Transfer, internalisation and retention of social and cognitive skills are inadequate because of insufficient organisational focus on transferring social and cognitive skills.

**Conclusion:**

Findings suggest a need for a broader and more profound focus on transferring social and cognitive skills to competency in clinical practice. Involving local ambassadors and increased collaboration between simulation centres and organisations around the transfer phase could optimise social and cognitive skills transfer. However, further research is needed in this area.

**Trial registration:**

N/A.

## What this study adds

- To engender competency among health professionals, transferring social and cognitive skills demands a triple focus on individual, intercollegiate, and organisational learning in the transfer phase.
- Transferring newly trained social and cognitive skills from simulation-based training to competency in clinical practice needs organisational effort and support to succeed.
- Transferring social and cognitive skills demands awareness and a mutual way of talking about social and cognitive skills among health professionals and management.
- Technical skills training focuses on individual learning, but social and cognitive skills depend on individual learning, intercollegiate learning and organisational learning, and so must the transfer process.
- Transfer of social and cognitive skills demands increased collaboration around the transfer process between the simulation-based education faculty and the management in clinical practice.

## Background

This article presents the findings of a study into health professionals (HP) transfer of social and cognitive skills^1^ (SCS) [1] from simulation-based training (SBT) to competency^2^ [2] in complex clinical practice.

It is known that SBT can increase knowledge and skills among pregraduate healthcare students [3, 4], SBT of technical skills places the participants higher on the learning curve when performing in clinical practice [5, 6], and participants can improve their SCS through SBT [5, 6]. Further, it is known that appropriate SCS can reduce adverse events [7, 8]. To reduce the number of adverse events in healthcare, SBT is increasingly used to teach, train, and maintain the knowledge and skills of qualified in-hospital HP worldwide [9–11]. However, there is a knowledge gap about how HP transfer new and trained SCS from SBT to competency in clinical practices.

Transfer is defined as the application of learning from SBT into clinical competency in the participants’ everyday; that is, what HP learns in one setting can be used in another comparable but different setting [12, 13]. To change or implement new or trained skills in clinical practice requires a transfer process, a social process involving more than knowledge and cognitive processes [14]. Moreover, SCS is embedded in one’s personality and cultural background [15]. Without a transfer process, focusing on meaning, motivation or conscious attention, the new or trained skill(s) will diminish, and the HP will return to usual habits and routines [16]. The relevance of researching skills transfer relates to the vast problems of patient safety [16, 17], global lack of HP, high personnel turnover, and well-being [18, 19].

**Fig. 1:**
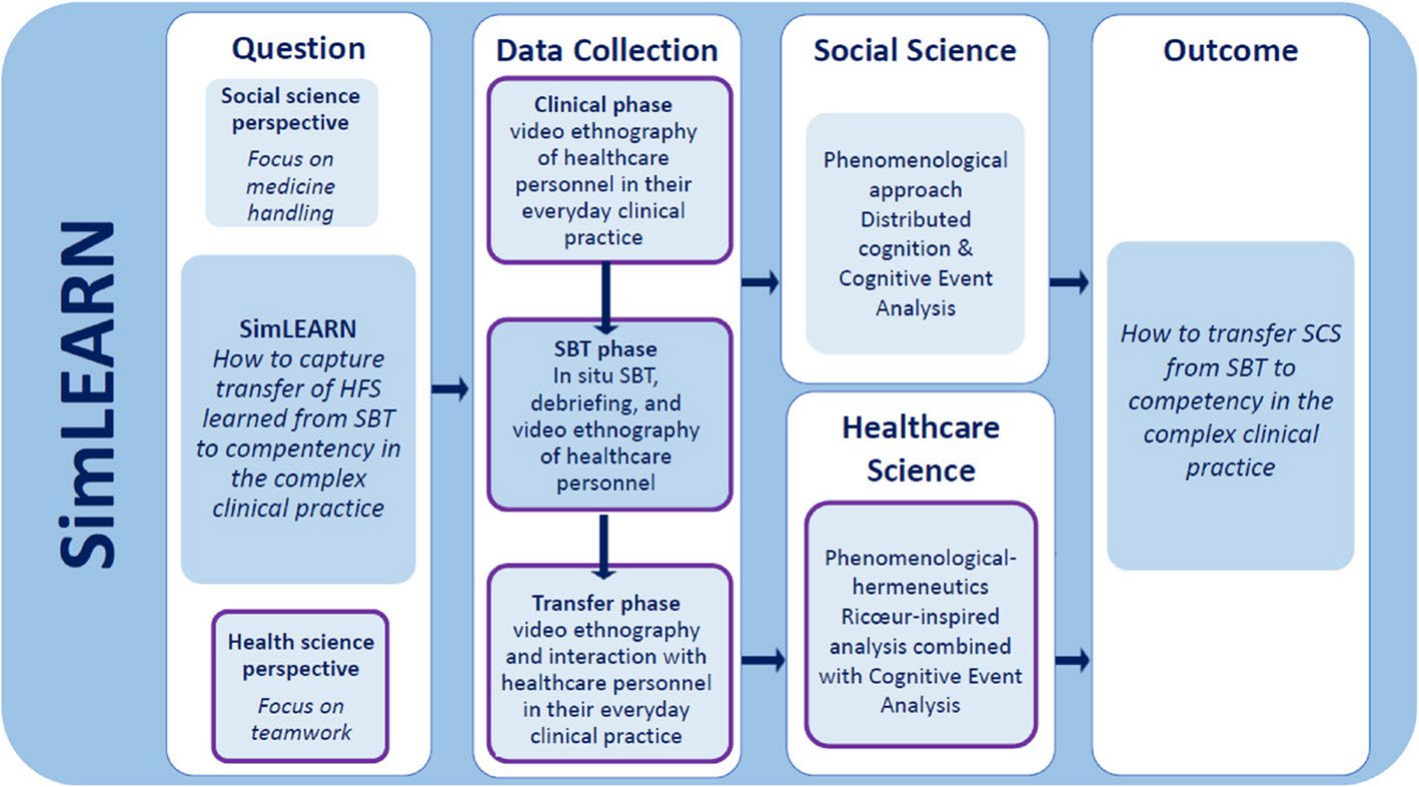
The SimLEARN study design

Given the gap in the research literature, developing new knowledge about how SCS knowledge becomes competency in clinical practice is crucial. The present study, therefore, investigated how transfer becomes competency in clinical practice after SBT. This study is part of the SimLEARN project (Fig. 1) that integrates a social science perspective [17] and a health science perspective on transfer of SCS with shared data. This paper presents the findings from the health science part of the SimLEARN, marked with a purple contour in Fig. 1.

### Aim

The aim of this study was to gain knowledge of how qualified HP transfers social and cognitive skills from an in situ simulation-based training course to competency in complex everyday clinical practices.

## Methods

### Study design

An ethnographic study using a qualitative phenomenological-hermeneutic methodology were used to investigate the transfer of qualified HP’s SCS before, during, and after an in situ SBT course. The didactic framework is based on the theory of adult learning [14, 18, 19], transformative learning [20, 21] and interprofessional education [22, 23]. The transfer of SCS to competency is based on Kolb, Bandura, Wahlgren, Billing, Dohn & Hachmann’s theories on learning and transfer [24–28]. The STROBE Statements are used as reporting guidelines [29].

### Ethnography

**Fig. 2:**
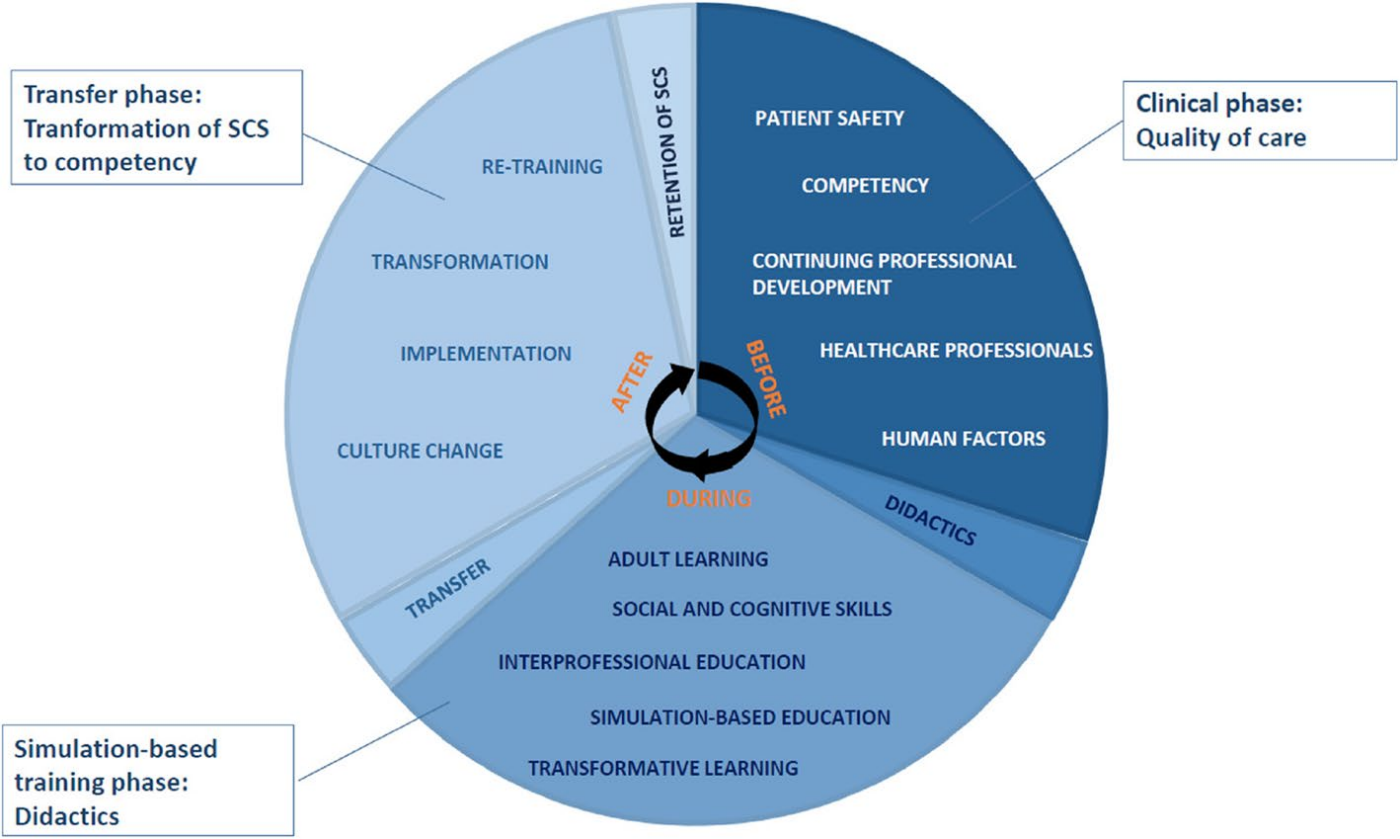
An overview and visualisation of the study’s theoretical frameworks

Based on the theory of participant observation [30] and organisational ethnography [31], two of the authors (LA and MLH) collected ethnographic data in three phases by shadowing HP with video cameras—observing their use of SCS in their work, and talking formally and informally—in their everyday clinical practice. The observations of SCS were based on the SCOPE framework [32]. The phases contained different focuses: *Clinical practice phase*intervention comprised preparatory information meetings. HP's SCS in clinical practice. *SBT phase*: progress of HP’s SCS during an in situ SBT course. After the first and second phases, an initial analysis focused on the following ethnographic phase. *Transfer phase*: how HP use SCS in their everyday clinical practice after participating in SBT (Fig. 2).

### In situ simulation-based training

The intervention comprised preparatory information meetings (~ 1 month before data collection begins), a screencast about SCS mailed to all HP (~ 1 week before SBT) and 2 days of SBT in each participating ward.

The first author (LA) developed the training scenarios based on the initial analysis of the clinical phase, from which SCS learning objectives were chosen. The learning goals differed a bit from ward to ward; however, in all four wards, the SBT circled, e.g. situational awareness (interruption, seeking help), decision-making (support, guidance, leadership, critical thinking), task management (plan and prepare, allocation of tasks), compassion (self-compassion, colleagues, patients and relatives) and teamwork (communication, co-coordination, responsibility). The scenarios were tested and validated by simulation operators and external facilitators. The facilitators had a facilitator course (~ EuSim level 1 and 2) and 2–10 years of experience as facilitators. The study objectives and methods were presented in a pre-training meeting with the facilitators. Further, the facilitators were aligned to the debriefing process, focusing on SCS, reflecting on the trained cases and making analogies to similar situations in the clinical practice.

The patient simulator used in the training course was Laerdal Medicals Nursing Anne Simulator (model 2019).

Each SBT day consisted of three high-fidelity training sessions, including a 5-min introduction to the simulator and the simulation situation, 10–20 min of in situ training, and a 25–30-min debriefing [33–36] led by the facilitators (1 doctor, 1 nurse). Four to six HPs participated in each scenario. The clinicians could participate in one to three of the scenarios. Two researchers (LA, MLH) observed, and three cameras recorded the training and debriefing from different positions.

After the 2 days of in situ simulation, the wards’ participants and heads (the daily leaders in the ward) were responsible for continued training and internalising the newly trained SCS. The heads were provided with a comprehensive list of the recently gained skills, attention points, and suggestions for further training after the SBT course.

### Settings

The data was collected in four different wards at two Danish hospitals—a university hospital (965 beds, ~ 11,000 personnel) and a local hospital (302 beds, ~ 2600 personnel). The included wards are two ICUs (54 beds, 8 beds), an emergency ward (42 beds) and an infectious disease ward (15 beds).

### Participants

The participants were at-work clinical HP (doctors, nurses, physiotherapists, radiologists, nurse assistants, medical students, nursing students, technicians and secretaries). The participants were selected from the duty schedules on the days of data collection and the SBT course to match a realistic everyday team. Thus, the competency on the day was similar to the expected span from beginner to specialist (five stages of expertise: novice, advanced, competent, proficient and expert) [37, 38]. However, all HP could opt to decline participation. Participants gave informed consent.

### Analytical approach

**Fig. 3:**
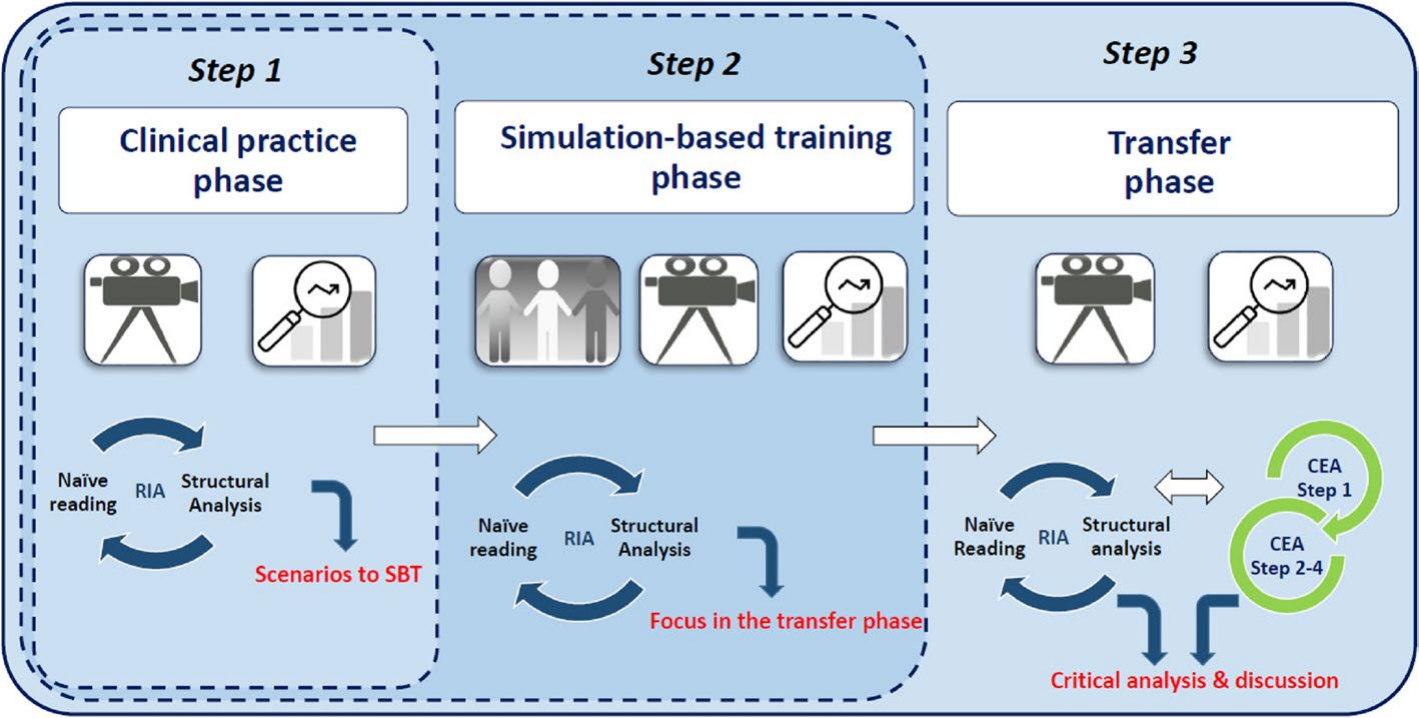
RI-CEA

The complexity of investigating changes in human behaviour called for a qualitative method comprising at least (1) a theoretical framework integrating social, psychological and cognitive aspects of performance, (2) an investigation of how SCS is taught, trained, learned and transferred, and (3) approaches to describe, understand and explain how SCS transfers into clinical competency. Therefore, a hybrid of two methods was designed where a Cognitive Event Analysis (CEA) was integrated in a Ricɶur Inspired Analytical approach (RIA) (Fig. 3). The hybrid method is called RI-CEA [39]. Data were initially analysed with RIA immediately after the clinical and SBT phases to incorporate the findings and focus on the following phase. After the transfer phase, all data were analysed as one complete dataset using RI-CEA. The CEA analysis was incorporated in the RIAs’ structural analysis, as demonstrated in Fig. 3, step 3.

## Findings

Data were collected between February 2019 and February 2020. The data consisted of video recordings, field notes, and reflections between the two researchers (LA, MLH). The data collection resulted in approximately 107 h of ethnography of HP’s teamwork.

**Fig. 4:**
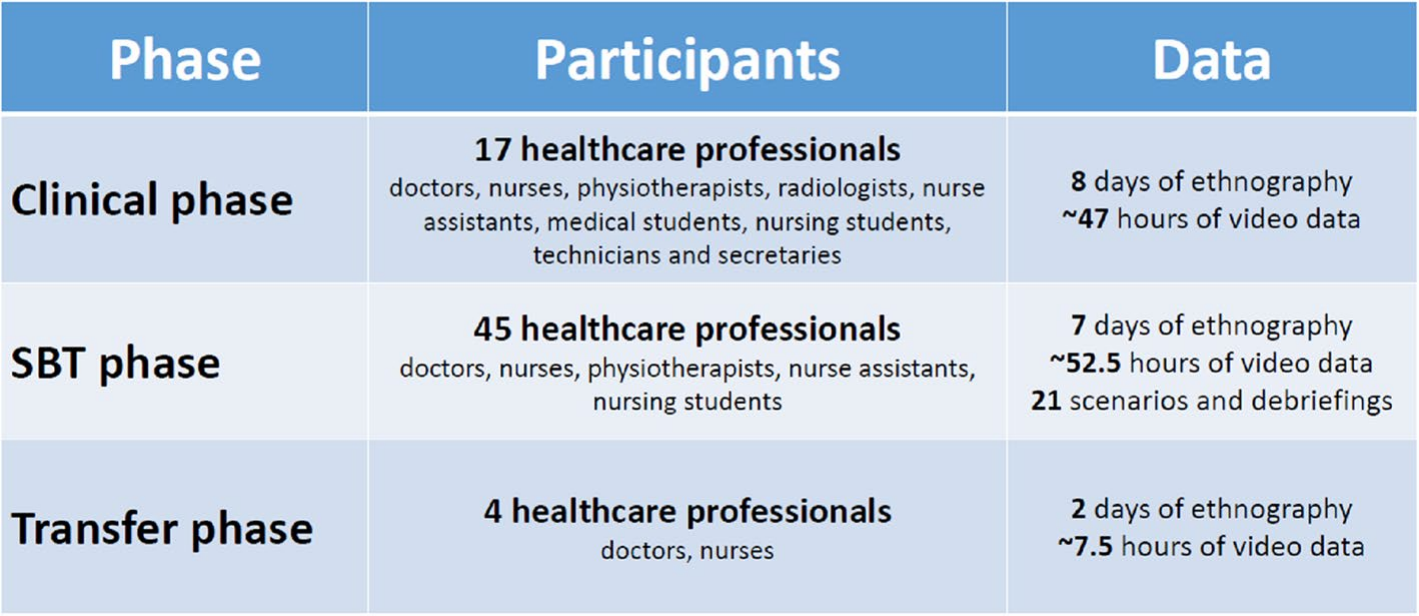
Overview of the study's included participants and data material

*The clinical phase* was completed within three months. Two days of ethnographic fieldwork were achieved in each participating ward (8 days total, 17 HP), equalling ~ 47 h of video data and field notes (Fig. 4). The subsequent analysis led to six themes: coordination, interruptions, educational responsibilities, teamwork and situational awareness, which were integrated into the SBT training course.

*The SBT phase* was completed within three months. Seven days of in situ training (21 scenarios and debriefings) were held (~ 52.5 h of video data) in four wards. Four expert facilitators (two doctors and two nurses) completed all the debriefings. Forty-five HP participated in the SBT courses. Due to a lack of HP and resources to participate, only one training day was completed in the infectious disease ward.

The subsequent analysis showed the immediate learning and training outcomes and, thus, nine themes: psychological safety, educational responsibilities, professional back-and-forth, teaching and learning during work, feedback from colleagues and SCS, leadership, teamwork, situation awareness, decision-making, *and task management.*

*The transfer phase* was cut short due to the COVID-19 pandemic. Only 2 days (4 nurses) of ethnographic fieldwork (7.5 h of video data) were completed at the local hospital, the ICU and the emergency department.

**Fig. 5:**
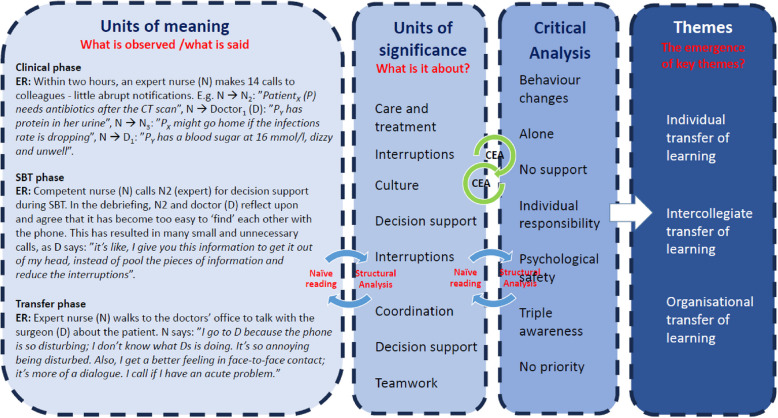
Ricɶur Inspired and Cognitive Event Analysis (RI-CEA)

After data were gathered, the RI-CEA analysis of the complete data set began. As shown in Fig. 5, RIA moved dialectically between parts (units of meaning and significance) and wholes (themes), between observations and statements. CEA explored real-time behaviour dynamics and took third-person macro-to-micro perspectives of the units of significance by identifying the cognitive result (i.e. joint decision-making) and working backwards to understand what caused the outcome. Finally, an integrated critical analysis and discussion of the findings were completed, resulting in three key themes, which are expanded below.

### Themes and subthemes

**Fig. 6:**
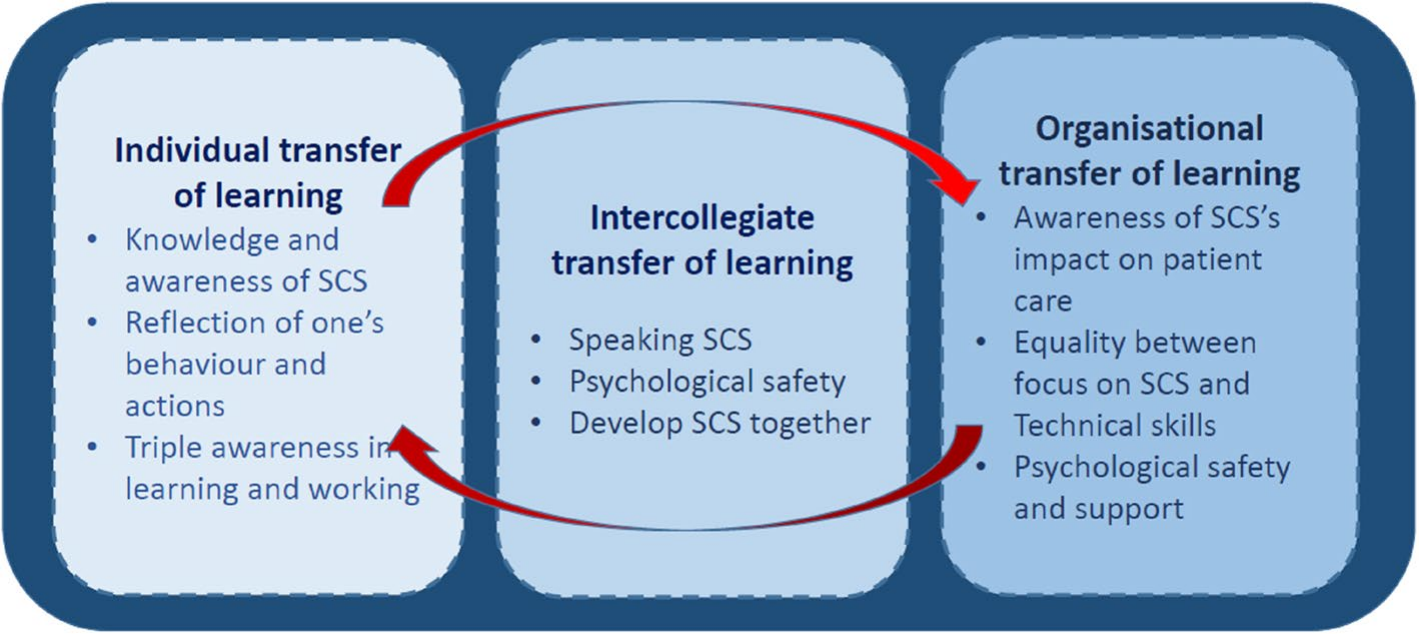
Findings, themes and subthemes that emerged through the Ricɶur Inspired and Cognitive Event Analysis (RI-CEA)

The analysis revealed three key themes: (1) individual transfer of learning, (2) Intercollegiate transfer, and (3) organisational transfer of learning. Each key theme has subthemes. The themes are intertwined and mutually dependent, but have different perspectives and content. The findings, themes and subthemes that emerged through RI-CEA, as shown in Fig. 6, will be elaborated on in the following.

### Individual transfer of learning

The characteristics of this theme were that the individual HP had an immersive role and obligation in integrating new SCS as a competency in everyday clinical practice. They experienced personal responsibility for integrating new knowledge from the SBT course into their everyday clinical practice. A nurse said: “It [transfer] is not something we talk about or address after a course; it’s solely my responsibility.”

This theme had three subthemes: (a) knowledge and understanding of SCS, (b) reflection on one’s behaviour and actions, and (c) triple awareness in learning, teaching and working.

####  Knowledge and awareness of social and cognitive skills

The knowledge and awareness of SCS before the SBT course were mainly limited to the concept of, e.g. ANTS (Anaesthetists’ Non-Technical Skill) [40, 41] and the importance of SCS in acute situations. Several participants highlighted in the debriefings and informal talks that SCS was not a topic or a focus in the clinical practice: “… only if it’s a critical situation.” The clinical practice was primarily focused on technical skills and implementation of algorithms, new medical procedures, and the use of new utensils or tools.

Reflections on how the participants looked at interruptions and how they interrupted others was a frequent topic, and several HPs became aware of their pattern of interruptions. The participants needed to flip the perspective away from their agenda and reflect on alternative behaviour. In a debriefing, an expert nurse said: “It’s interesting to reflect on our interruption culture [situation-awareness]. Only now, I understand that SCS are more than closed loops and ABCDE and that it also influencesour everyday work.” In the transfer phase, the same nurse said: “After the SBT course, I realised that my workday is one long interruption. I have tried to change my habit of interrupting others. However, I end up doing as usual [routine].” These two quotations showed that this nurse had become aware of SCS, gained knowledge about SCS and its impact on her work, and tried to transfer the awareness into her complex clinical everyday.

#### Reflection of one’s behaviour and actions

Most participants declared that the SBT course offered a platform for reflecting upon their behaviour and actions. In the transfer phase, the reflection of one’s actions was observed when a competent nurse thought out loud in her teamwork with another nurse when preparing to transfer her patient from the ICU: “… if I do this first, then we have more room for that [informing the patient] later.” Asking the nurse why she did that, she said that the SBT made her aware that by coordinating, they saved time in a busy everyday and that she forgot fewer things after coordinating with a colleague. A competent doctor said in a debriefing, “The focus solely onSCS in the talks [debriefing] made me look inward on my behaviour. I couldn’t hide behind my medical knowledge; I had to check my side of the interactions. Now I understand and am trying to become a better team player.” The findings indicated that the SCS focuses on reflection about one’s behaviour and actions, which could lead to an insight into one’s role in the interaction. HP needs more than medical knowledge.

#### Triple awareness in teaching, learning and working

A triple awareness—balancing learning, teaching and working—was observed across all three phases. Learning, teaching, and working are not SCS; however, keeping one’s balance between the work of learning, teaching, and taking care of the patient simultaneously is a profound cognitive SCS, including critical thinking, decision-making, and problem-solving. HP performed most of their work in this triple role of teaching, learning, and caring for patients and relatives. Several HP said they did not learn this skill in their education or as newly hired; they taught themselves to combine work with learning and implementing individually developed SCS.

An advanced nurse said, “Although I have worked here for 1½ years and amcompetent, I must teach my new colleagues, care for patients, and seek guidance from the expert nurses. Nevertheless, sometimes I justwant to work without integrating new stuff.” This quote described that HP must cope with this triple awareness to transfer new knowledge, reflect on and be ready to change behaviour and actions. Moreover, an expert nurse expressed that since the SBT, she has considered whether she should start teaching her new colleagues and students about this triple awareness. However, she felt she lacked the competencies to do so.

Data showed an insignificant change for some HP from the clinical phase to the transfer phase. A competent doctor stated that through the SBT, he became aware of this complex task and tried to find a better way to balance his roles. An expert nurse expressed that she had taught herself to manage the balance of her triple role: “Nobody has ever talked about thistriple role or taught me how to do it; it’s just how it is. It makes sense; working here is like that.” This finding suggests that HP were unaware of the more hidden SCS, although these significantly impacted their everyday work.

The analysis of this key theme revealed that if transfer of SCS from SBT to clinical practices should succeed, the individual must be ready to challenge their routines and behaviour, use the new skills, and deal with the constant need for triple awareness. In their own words, SBT helped them become aware of hidden SCS.

### Intercollegiate transfer of learning

The characteristics of this theme were the intercollegiate responsibilities in training the new or changed behaviour and actions among colleagues after the SBT course. An example from the transfer phase showed this training in the clinic: two nurses walk towards each other in the hallway. An advanced nurse (N1) smiles at an expert nurse (N2), who slows and nods to N1. They s*top and begin to coordinate and engage in joint decision-making.* They participated together in the SBT course. The nurses looked as if they communicated silently with their bodies and faces. When N1 smiled, N2 slowed down, and her nod was an invitation to N1 that she may interrupt N2. They did not coordinate with words but interpreted each other’s embodied behaviour.

The theme was divided into three subthemes: (a) Speaking SCS, (b) Psychological safety, and (c) developing SCS together.

#### Speaking social and cognitive skills

The participants gained awareness and new ways to speak about SCS. A doctor and a nurse agreed: “… we’ve talked about it [SCS] after the course and try to support each other to improve it, … but never with others; … it’s difficult because they weren’t there [in the SBT debriefings].” This specified that they gained awareness and a way of talking about SCS, which they shared in close collegial relations, and that the SBT made them aware of the significance of SCS. Nevertheless, they shared this awareness and way of talking about SCS with colleagues they trained with, not broadly in the ward or with the managers. SCS thus became a distinctive skill for some HP. CEA showed this multiple times.

#### Psychological safety

The participants mentioned the need to feel safe and secure in SBT, debriefings, and experiments using the new or changed behaviour in practice. An expert nurse declared: “I only dare if I feel secure …tryingthe new stuff, you know, without feeling anxiety and the senseof being exposed or judged.” Some articulated that they considered SBT a privilege to train as a team and improve as a benefit for the patients. However, there were some barriers in the clinical setting. The colleagues primarily spoke with those whom they felt safe.

Some participants mentioned a mutual understanding that good SBT, debriefings and transfer demand an open feedback culture in the ward. However, it was a challenge “… when the leaders don’t show the way”, “… if I don’t feel safe among my colleagues,” or “… when the personnel flow isthis big.” This indicated that HP wanted to use and transfer the newly learned, but struggled to succeed.

#### Developing social and cognitive skills together

The professional roles developed and became competency through interactions with colleagues, from novices to experts. In the SBT, a competent nurse (N1) received a delirious, acute, sick patient. The competent nurse called an expert nurse (N2) to the room. N2 could have taken the lead but instead supported N1 in her leadership. N1 tells in the debriefing that she, simultaneously with the coordination and teamwork with the doctor, observes how N2 acts in the situation: “Because I hope to become an expert like her.” This example demonstrated that HP learned and taught how to do effective teamwork when working as a team and that the less experienced gained support, a role model and experiences through their work. A doctor expressed: “Simulation is one of the only places where we learn and reflect across the interdisciplinary barriers, and this makes us better as a team in difficult situations, not only the acute.” 

HP expressed, across the data, that current SCS (SCOPE) training is primarily an add-on to courses aiming at highly acute situations and algorithms. In this study, HP gained a new understanding of SCS through the three phases. Nevertheless, HP’s speech about SCS changed only among the SBT participants. From talking about SCS as closed-loop, teamwork and leadership, they said in the transfer phase about different ways of being aware in other situations, working with interruptions and balancing learning, teaching and working.

The analysis of this key theme expressed that if transfer of SCS from SBT to clinical practices should succeed, the colleagues must have a mutual awareness and a way to talk about SCS and keep practising, reflecting and supporting each other in the transfer process. This work demands psychological safety among HP, which is necessary for internalising the knowledge in the individual self and the ward.

### Organisational transfer of learning

This theme’s characteristics were organisational awareness and a focus on transferring SCS in areas other than acute situations. All participants expressed differently that the organisational support for the newly trained SCS transfer has yet to be adopted. A nurse said, “No one asked me what I’ve learnt or need to implement … I’m on my own.” The organisation seemed to lack focus on implementing knowledge of SCS to become a competency.

This theme was divided into three subthemes: (a) awareness of SCS’s impact on patient care, (b) equality between SCS and Technical skills and (c) psychological safety and support.

#### Awareness of social and cognitive skills impact on patient care

The findings implied a lack of organisational awareness towards SCS. In the transfer phase, competent and expert participants disclosed that when they do mandatory training, they train in acute and rare situations using different SCS tools, mainly focusing on leadership and communication (SBAR and Closed-loop). Moreover, they expressed that transfer of SCS in clinical practice is rarely focused on after a course. An expert nurse said: “Sometimes the heads [leaders] underline the importance of SBAR and Closed-loop during clinical meetings. However, it’s my responsibility to know how to change my routines, request it from my colleagues and teach it to the new ones.” The quote demonstrated the absence of awareness of the necessity of focusing on transfer after a course to integrate the new skills into competency. Furthermore, HP expressed that the workload and the individual responsibility of transferring the new skills to competency induced them to return to their usual routines and behaviour.

#### Equality between social and cognitive skills and technical skills

Doctors and nurses mutually disclosed that there had been no organisational focus on SCS after the course, either in memos or meetings. HP had yet to hear which SCS the ward should implement or train further. In contrast, both wards in the local hospital focused on implementing technical skills, such as using a new patient relaxing chair and a new machine to test blood samples. This indicated that technical skills were prioritised over SCS.

#### Psychological safety and support

More participants expressed a need for support from the organisation to keep them motivated during the transfer process. A nurse said: “When no one cares if I do it or not, why then use the energy?everydayis busy as it is”. Further, the findings indicated a deficiency in psychological safety as the HP only spoke with those they trained.

The analysis of this key theme showed that the wards involved in the project had yet to support the transfer of SCS at an organisational level despite the material given to the heads. The findings suggested that if transfer of SCS from SBT to clinical practices should succeed at an organisational level, the management must (in parallel with HP) gain awareness of SCS and increase their focus on SCS transfer. This is equally important as new guidelines, tools and procedures.

## Discussion

Transfer from SBT to competency depends on three intertwined levels: *an individual, an intercollegiate and an organisational level.* This finding was consistent with existing research about transfer of learning, internalising, and retention of new skills [13, 42–45]. However, transfer and retention of SCS are different from transfer of knowledge. SCS are, nevertheless, also embedded in the individual’s personality, depending on one’s history, culture and characteristics.

**Fig. 7:**
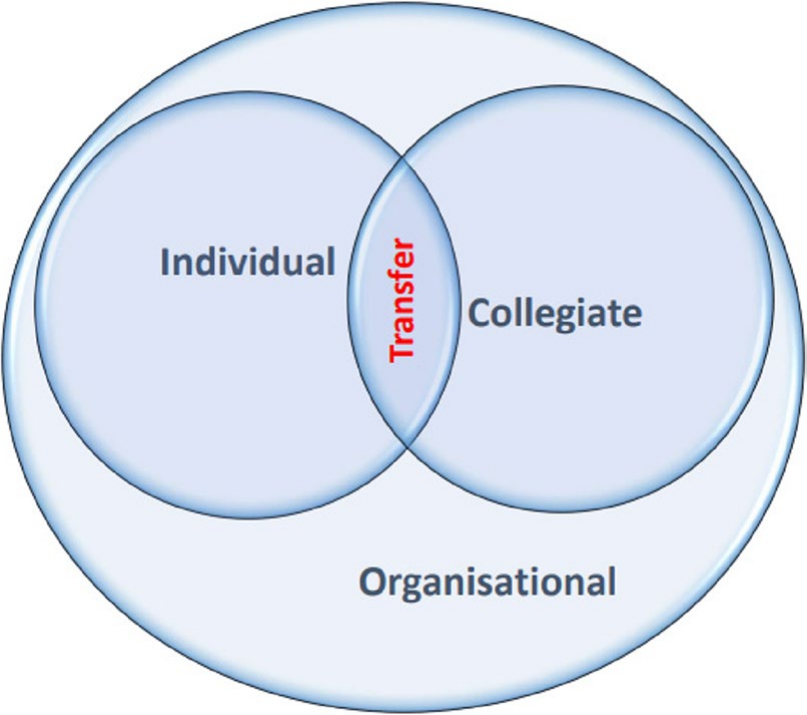
Transfer of social and cognitive skills

Integrating individual, intercollegiate and organisational processes is necessary to gain transfer of SCS, as shown in Fig. 7. In the transfer process, the individual must be ready to learn, reflect upon their actions, and feel psychologically safe to integrate the new competencies into their actual behaviour, as noted in diverse didactic literature [21, 25, 46–48]. Likewise, the intercollegiate must articulate SCS among each other to make SCS explicit and execute constructive feedback, which also demands psychological safety, a SCS language, group reflections and courage [49, 50]. The organisation surrounds the individual and the intercollegiate in the transfer process. The organisation is HP’s frame and condition; thus, the organisation must take the lead in the transfer process, nudge HP, and actively empower and support the individual and intercollegiate transfer process to complete a successful implementation [51]. The three aspects of transfer are discussed separately below.

### Individual transfer

The findings revealed behavioural changes in SCS among individual SBT participants compared to the clinical phase. As Dohn and Markauskaite [45] describe, individual competency develops through transfer, resituation, and transformation processes that require adapting knowledge across contexts. Successfully transferring SCS into clinical competency demands readiness to change routines, along with awareness, reflection, and a grasp of the language and meaning of SCS [52]. Managing the “triple awareness” of learning, teaching, and working emerged as a persistent individual challenge for HPs, one complicated by external demands from management and policy. This triple awareness may obstruct the transfer process. A readiness for self-directed, lifelong learning [47] appears crucial, as it allows HPs to navigate the complexity of integrating SBT into everyday practice. Adults learn when prepared, can relate learning to their roles, and find relevance and safety in the process [18]. This was reflected in the data—for instance, participants’ shared understanding that reducing interruptions, an SCS element, could enhance patient safety and HP well-being.

### Intercollegiate transfer

The transfer of situational clinical skills (SCS) was influenced not only by individual factors but also by intercollegiate relations, organisational culture, and psychological safety [49, 53]. Although behavioural changes in intercollegiate SCS were observed after SBT, these were most evident among course co-participants. The CEA microlens highlighted successful transfer when comparing post-course interactions with similar pre-course situations. As Elkjaer [52] asserts, learning is a social process of action and reflection, where individual skills must evolve into shared practices. Wenger [47, 54] similarly underscores the role of communities of practice in adult learning, reflected in how course participants more readily engaged in SCS-related reflection during debriefings.

Workplace culture played a significant role, requiring shared language and recognition of SCS as vital to teamwork and patient safety [53, 55–58]. Reflections and discussions around SCS occurred primarily among those who had attended the course together, suggesting psychological safety and mutual understanding as key enablers. Edmonson and others have shown that such safety is critical for facilitating peer feedback and behavioural change [49, 59]. These findings suggest that individual and intercollegiate transfer processes are deeply interwoven, reinforcing each other. Strengthening communities of practice and fostering psychological safety may therefore be essential strategies to support sustained transfer of SCS into clinical settings.

### Organisational transfer

The transfer of SCS from SBT to clinical practice requires deliberate organisational effort, yet this study revealed a lack of structured follow-up and focus. Despite significant investment in training, hospitals often fail to embed SCS into daily routines [60]. As Yamnill and McLean note, “learning is of little value to organisations unless it is transferred in some way to performance” [42, p.196], highlighting the importance of institutional responsibility in turning individual learning into collective competence.

No systematic implementation of SCS was identified in the participating wards post-training. Technical skills appeared to receive more attention, likely due to their tangible and individually measurable nature. In contrast, SCS are social and context-dependent, requiring collaboration, reflection, and organisational support for effective transfer [51, 56]. A common misconception is that SCS are innate or only relevant in emergencies. However, the data suggest that SCS can be taught and integrated if made explicit and continuously reinforced through reflective practice.

Leadership is key in this process. Rather than viewing course completion as sufficient, leaders must ensure structures are in place to sustain behavioural change. This includes creating space for reflection and learning and recognising SCS as essential to patient safety. Without such scaffolding, transfer remains isolated, and the potential impact of SBT is lost.

In theory, this study’s findings mean that it is possible to show how and if the transfer of newly trained SCS happens, although it is time-consuming. This study could form the basis of further research on transferring SCS and develop more straightforward ways to increase the focus on transfer. The findings mean that faculty planning a course must emphasise the organisational aspect of transferring SCS in clinical practice; the learning continues after the debriefings and is transformed into competency. The transfer to competency can succeed through increased collaboration with the managers in clinical practice about who, what, and how learners can continue the training and intensify the implementation in the clinical practice. This study revealed the importance of involving all three levels (individual, intercollegiate and organisation) in transferring and implementing new learning, knowledge or equipment.

### Limitation

This study has some limitations that need to be mentioned. First, due to COVID-19, data from the transfer phase was only collected from two wards instead of four and only from the small local hospital, in the ICU and emergency department. The findings could differ if transfer data were collected at all four wards. On the other hand, data from the clinical and SBT phases were similar, making it possible that data from the transfer phase would be consistent, strengthening the credibility of the findings. Second, the Hawthorn Effect represents a potential bias [61] in behaviour alteration due to observation. Third, there was a risk of potential selection bias because the SBT course was not mandatory. The Hawthorn effect and the possible selection bias weaken credibility because the participants could act differently if they were watched. They accepted SBT as a learning method, and it cannot be assumed that their experiences, reflections and actions are representative [62]. Nevertheless, video, physical and cognitive responses from the participants and fieldnotes reduce this bias as they allow going back and forth in the research group's situations and discussions*.* Fourth, the hybrid method has an in-build validation, as the researcher has to go back and forth between units and the whole to verify if the assumptions stand. Further, analysis workshops were held with the research team throughout the analytical process to reduce misinterpretations and overinterpretations, increasing dependability. The findings’ transferability is high because data are gathered in four different settings and environments. Also, these findings can be used in all kinds of transfer from teaching to competency. Finally, the data overrepresents nurses, minimising the insights into other HP’s transfer.

## Conclusion

This study suggests that SBT of SCS can be transferred to competency in clinical practice. However, further focus on the organisational role and responsibility in implementing the SCS is needed if the competency should be more than a detached individual skill.

Findings suggest that SBT allows participants to talk about SCS and how to use SCS in their everyday clinical practice. There is a need to focus more on transfer to integrate the newly trained into competency in clinical practice and develop organisational learning by including clinical leaders. New SCS from SBT only leads to competency in clinical practice if a transformation plan and daily focus on using the new skills are carried out. Still, a more organisational view on training events is necessary if the competency is to become a culture rather than an individual skill.

More research on transfer to competency is necessary by executing follow-up fieldwork with participants, for instance, after a week, a month, and 3 months after the SBT.

## Data Availability

The data are protected by the participants'privacy and can, therefore, not be shared openly. The data are, however, available from the corresponding author upon reasonable request and with the permission of the ethical committee and The Danish Data Protection Agency.

## Abbreviations

ANTS: Anaesthetists’ Non-Technical Skills
CEA: Cognitive Event Analysis
SCS: Social and Cognitive Skills
RIA: Ricɶur Inspired Analytical approach
RI-CEA: Hybrid method of a Ricɶur Inspired Analytical approach and Cognitive Event Analysis
SBT: Simulation-based training

## Footnotes

1 Social and Cognitive skills: include communication, interpersonal, collabration and decision-making skills and puts emphasis on how the environment, the organisation and human psychology interact (1).

2 Competency is defined as a skill developed on basis of knowledge, experiences and behaviour towards qualified activities that can be put into action in everyday practice. Competency demands actions, which are both intentional and directed, as well as individual, personal and subjective. This definition is inspired by the Danish pedagogic term “action competencies” (2).
